# Inositol Polyphosphate 4-Phosphatase Type II Is a Tumor Suppressor in Multiple Myeloma

**DOI:** 10.3389/fonc.2021.785297

**Published:** 2022-01-05

**Authors:** Yafei Wang, Lin Chen, Qian Li, Shuang Gao, Su Liu, Jing Ma, Ying Xie, Jingya Wang, Zeng Cao, Zhiqiang Liu

**Affiliations:** ^1^Department of Hematology, Tianjin Medical University Cancer Institute and Hospital, National Clinical Research Center for Cancer, Key Laboratory of Cancer Prevention and Therapy, Tianjin’s Clinical Research Center for Cancer, Tianjin, China; ^2^Department of Hematology, Tianjin Cancer Hospital Airport Branch, Tianjin, China; ^3^Department of Physiology and Pathophysiology, School of Basic Medical Science, Tianjin Medical University, Tianjin, China

**Keywords:** multiple myeloma, INPP4B, cell proliferation, PI3K/Akt, tumor suppressor

## Abstract

Inositol polyphosphate-4-phosphatase type II (INPP4B) has been identified as a tumor suppressor, while little is known about its expression and function in multiple myeloma (MM). In this study, we evaluated the expression of *INPP4B* in 28 cases of newly diagnosed MM patients and 42 cases of extramedullary plasmacytoma (EMP) patients compared with normal plasma cells and found that low INPP4B expression was correlated with poor outcomes in MM patients. Moreover, expression of INPP4B in seven MM cell lines was all lower than that in normal plasma cells. In addition, loss of function of INPP4B promoted cell proliferation in MM cells; however, gain of function suppressed MM cells proliferation and arrested the cell cycle at G0/G1 phage. Meanwhile, knockdown of INPP4B enhanced resistance, but overexpression promoted sensitivity to bortezomib treatment in MM cells. Mechanistically, we found that *INPP4B* exerted its role *via* inhibiting the phosphorylation of Akt at lysine 473 but not threonine 308, which attenuated the activation of the PI3K/Akt/mammalian target of rapamycin (mTOR) signaling pathway. Therefore, we identified an inhibitory effect of INPP4B in MM, and our findings suggested that loss of INPP4B expression is a risk factor of aggressive MM.

## Introduction

Multiple myeloma (MM), which arises from the uncontrolled proliferation of malignant plasma cells, is the second most common hematological malignancy (1). The overall survival (OS) of MM patients has been significantly increased due to the availability of drugs such as immunomodulatory drugs (IMiDs), proteasome inhibitors (PIs), and monoclonal antibodies, and transplantation of autologous stem cells (2–4). However, MM is still incurable, and almost all patients will suffer from disease recurrence (5). Therefore, it is of great importance to further elucidate the mechanisms underlying MM progression in order to develop more effective therapies.

The advances achieved recently shed new light on the pivotal role of inositol polyphosphate 4-phosphatase type II (INPP4B)-mediated signaling in the regulation of cancer survival, motility, and invasiveness (6). The human *INPP4B* gene is located on chromosome 4q31.21 and encodes inositol polyphosphate 4-phosphatase II, which is widely expressed in many human tissues, with the highest expression in the skeletal muscle and heart and relatively low expression in the brain (7). INPP4B is a phosphoinositide phosphatase that plays complex and controversial roles in the pathogenesis of different tumors (8). Several studies have proven that INPP4B expression was inhibited in digestive cancers, acute myeloid leukemia, melanomas, and breast cancer due to the deletion of the *INPP4B* chromosome region (9), and served as an oncogenic factor (10–13). Recently, increasing evidence showed that INPP4B is a negative regulator of the PI3K/Akt signaling pathway in many cancers (14). INPP4B inhibited PI3K/Akt signaling pathway suppresses PI3K/Akt signaling by converting PI(3,4)P(2) to PI(3)P (15, 16). Furthermore, loss of INPP4B protein expression in breast and ovarian cancer is associated with decreased patient survival (17). The PI3K/Akt signaling pathway and the mammalian target of rapamycin (mTOR) signaling pathways are two pathways crucial to many aspects of cell growth and survival in cancers (18), and a recent study has revealed that INPP4B suppressed the PI3K/Akt/mTOR signaling in cervical cancer cells (14). These observations raise the possibility that INPP4B might affect PI3K signaling and function as a tumor suppressor, while the exact role of INPP4B in MM is unknown.

In the current study, we aim to explore the expression of INPP4B in MM clinical specimen and cell lines and study the clinical significance of INPP4B and its role in regulating PI3K/Akt/mTOR signaling pathway.

## Materials and Methods

### Patients and Samples

Twenty-eight patients at the Tianjin Medical University Cancer Institute and Hospital between January 2014 and January 2017 were enrolled in this study. Written informed consent for publication of their clinical details was obtained. All 28 enrolled patients met the International Myeloma Working Group (IMWG) criteria. The clinical and laboratory data were reviewed in retrospect, and data collected included patient gender, age, subtype, clinical stage, serum lactate dehydrogenase (LDH), β2-MG, albumin, creatinine, and Ca^2+^ levels. Overall survival (OS) was calculated from the first date of diagnosis until death from any causes or until the date of the last contact for surviving patients. Progression-free survival (PFS) was defined as the date from diagnosis to the first occurrence of disease progression, relapse after response, or death due to myeloma. Primary CD138+ plasma cells were isolated from bone marrow aspirates of either healthy donor or MM patients by Ficoll–Hypaque density gradient sedimentation followed by antibody-mediated positive selection using anti-CD138 magnetic-activated cell separation microbeads (Miltenyi Biotec, Germany).

Forty-two patients who presented with extramedullary plasmacytoma (EMP) at the Tianjin Medical University Cancer Institute and Hospital between January 2011 and January 2016 were enrolled in this study. The tumor samples used in this study were all derived from EMP that were independently identified by two expert pathologists.

Key exclusion criteria include other hematological malignancies such as lymphoma, leukemia, myelodysplastic syndrome, and all kinds of solid tumors, and patients who received prior treatment with IV bisphosphonates, planned radiation, or surgery to bone.

### Cell Culture

Human MM cell lines, LP-1, OPM-2, RPMI 8226, U266, H929, MM.1S, and MM.1R myeloma cells were generously provided by the Institute of Hematology and Blood Diseases, Hospital Chinese Academy of Medical Sciences and Peking Union Medical College. LP-1, OPM-2, RPMI 8226, H929, MM.1S, and MM.1R were maintained in Roswell Park Memorial Institute (RPMI) 1640 medium and supplemented with 10% fetal bovine serum (FBS) in 5% CO_2_ at 37°C. U266 was maintained in RPMI 1640 medium and supplemented with 15% FBS in 5% CO_2_ at 37°C. RPMI 1640 culture media were obtained from HyClone (Logan, UT, USA). FBS was obtained from Gibco (Grand Island, NY, USA). Other cell culture reagents were obtained from Bio-Rad Laboratories (USA). All cells were short tandem repeat (STR) authenticated (BioWing Biotech, Shanghai, China) and mycoplasma-free confirmed with the Universal Mycoplasma Detection Kit (ATCC, Manassas, VA, USA).

### Quantitative Real-Time PCR

Total RNAs from the primary patient MM cells and cultured cell lines were extracted using the Trizol reagent (Invitrogen, Carlsbad, CA, USA), and reverse transcription reactions were performed using PrimeScript RT Reagent Kit (TaKaRa, Dalian, China). Quantitative real-time PCR was then performed to amplify the cDNAs with the SYBR Green PCR kit on Biosystems 7500 PCR system. INPP4B expression levels were quantified using the 2^−ΔΔCt^ method. β-2M was used as the internal control. The primers for *INPP4B* were 5′-GGAAAGTGTGAGCGGAAAAG-3′ (forward) and 5′-CGAATTCGCATCCACTTATTG-3′ (reverse), and the primers for β-2M were 5′-TCTCTGCTCCCCACCTCTAAGT-3′ (forward) and 5′-TGCTGTCTCCATGTTTGATGTATCT-3′ (reverse).

### Immunohistochemical Staining

EMP histological sections were deparaffinized and blocked with 3% H_2_O_2_ solution, and antigen was retrieved with 10 mM citrate buffer (pH 6.0). After blocking, appropriately diluted primary INPP4B (#14543, Cell Signaling Technology) antibodies were added onto the slides and incubated in a humidified chamber at 4°C overnight, and then, appropriately diluted biotinylated secondary antibody was incubated at room temperature for 1 h. 3,3′-Diaminobenzidine (DAB) substrate solution (Dako, K5361) (freshly made just before use) was used to reveal the color of antibody staining. Nuclei were localized by hematoxylin staining for 1–2 min before mounting and capture.

### Western Blot Analysis

Total protein was extracted from cells in radioimmunoprecipitation assay (RIPA) lysis buffer and separated by 10% sodium dodecyl sulfate–polyacrylamide gel electrophoresis (SDS-PAGE) gel electrophoresis and then transferred to polyvinylidene fluoride (PVDF) membranes. The membranes were blocked in 5% non-fat milk in Tris-buffered saline with Tween 20 (TBST) for 1 h and incubated with primary antibodies against INPP4B (1:1,000), total Akt (1:1,000), phospho-Akt Thr308 (1:1,000), phospho-Akt Ser473 (1:1,000), mTOR (1:1,000), and Rictor (1:1,000) overnight at 4°C, followed by secondary antibodies for 1 h at room temperature. Primary and secondary antibodies were all purchased from Cell Signaling Technology. β-Tubulin and β-actin were used as internal control. The optimized time point of INPP4B overexpression and knockdown was confirmed according to our previous studies (19, 20).

### Cell Proliferation Assay

Cell proliferation of LP-1, RPMI 8226, and MM.1S cells after overexpression and knockdown was examined by CCK-8 assay. LP-1, RPMI 8226, and MM.1S cells were plated in 96-well plates at a density of 5 × 10^4^ cells (90 μl) per well. After 0, 24, 48, and 72 h, 10 μl of CCK-8 (Dojindo, Japan) was added into each well. After incubation for 2 h at 37°C, the plates were read at 450 nm with a Microplate Reader 550 (Bio-Rad Laboratories, Richmond, CA, USA). The following formula was used to calculate cell viability = OD value of overexpression group/OD value of control group.

### Transfection, Virus Package, and Infection

HEK293T cells in a 10-cm dish were transfected with PMD2G and PSPAX2 packaging plasmids (Addgene, Watertown, MA, USA), together with lentiviral-expressing vectors encoding target genes INPP4B (GeneCopoeia, Guangzhou, China). Supernatant carrying the viral particles was harvested after transfection. For viral infection, myeloma cells were seeded at (1−2) × 10^6^ cells per well in six-well plates and then added supernatant carrying the viral particles. Twelve hours after infection, the medium was changed, and cells were cultured for another 48 h until further management.

### Cell Cycle

To analyze the cell cycle distribution, cells were added supernatant carrying the viral particles for 12 h, and cells were cultured for another 48 h; then, 2 × 10^6^ cells were collected after 6 h of starvation. Cells were washed twice with phosphate-buffered saline (PBS), fixed in 75% ice-cold ethanol overnight, incubated with staining buffer (10 μg/ml RNase A, 50 μg/ml PI, and 4 mM sodium citrate) at 37°C for 10–30 min in the dark and then assessed by flow cytometry. Cell cycle profiles were analyzed using FlowJo 7.6 software.

### Flow Cytometry Assay

Annexin V-fluorescein isothiocyanate (FITC)/propidium iodide (PI) double staining kit (FITC-conjugated Annexin V) (eBioscience, USA) was used to label apoptosis cells. Briefly, 1 × 10^6^ cells were resuspended in 0.5 ml staining binding buffer, and then, Annexin V-FITC (5 μ) and PI (1 μl) were added to the cells, respectively. Cells were stained for 15 min at room temperature and subjected to flow analysis. FlowJo software was used to analyze the results.

### Statistical Analysis

Data were shown as mean ± SD for at least three independent experiments. All data analyses were performed using SPSS software version 21.0 (SPSS Inc., Chicago, IL) and GraphPad Prism 5. For overall survival (OS) and progression-free survival (PFS) assay, the patients were divided into a high-expression group and a low-expression group according to the median of INPP4B expression. *p*-values are presented as two-sided, where statistical significance was considered a *p* < 0.05.

## Results

**Figure 1:**
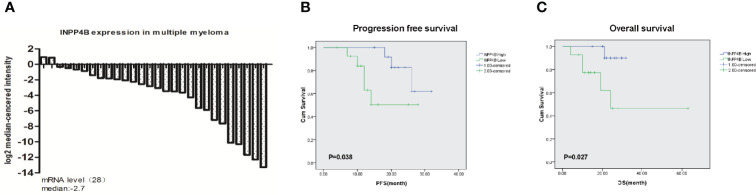
Expression of INPP4B in MM patient samples. **(A)** The expression level of INPP4B in newly diagnosed MM patient samples. **(B)** Correlation of INPP4B expression with progression-free survival (PFS) and **(C)** overall survival (OS) in patients with newly diagnoses MM patients.

To determine whether INPP4B has clinical significance in MM patients, we analyzed the expression in newly diagnosed MM patient samples. The major clinical characteristics of 28 MM patients enrolled in this study are shown in **Table 1**. The expression level of INPP4B in bone marrow plasma cells from 26 patients was lower than that of normal donors, and only two patients presented a higher expression level (**Figure 1A**). Twenty-two MM patients were enrolled for overall survival and PFS statistics. The follow-up time started from the date of diagnosis until December 31, 2019, or death, ranging from 1 to 68 months with a median of 25 months. The PFS and OS assays showed that the patients with lower INPP4B expression level had a worse survival rate (*p* = 0.038), and the patients with higher INPP4B expression had better OS rate (*p* = 0.027) (**Figures 1B, C**). Collectively, these data suggest that INPP4B plays a pivotal role in the clinic outcomes of MM patients.

**Table 1:** Clinical characteristics of 28 MM patients.

Baseline characteristics	n (%)
Age	
>65	11 (39%)
≤65	17 (61%)
Gender	
Male	20 (71%)
Female	8 (29%)
Type	
IgG	12 (42%)
IgA	7 (25%)
Light chain	9 (33%)
DS stage	
I	0
II	5 (17%)
III	23 (83%)
ISS stage	
I	6 (21%)
II	10 (35%)
III	12 (44%)
LDH	
Normal	3 (10%)
>Normal	25 (90%)
β2-MG (mg/L)	
<5.5	18 (64%)
≥5.5	10 (36%)
Albumin (g/L)	
<35	16 (27%)
≥35	12 (73%)
Creatinine (mmol/L)	
>177	2 (7%)
≤177	26 (93%)
Ca^2+^ (mmol/L)	
≤2.75	24 (86%)
>2.75	4 (14%)

DS, Durie–Salmon; ISS, International Staging System; LDH, lactate dehydrogenase.

**Figure 2:**
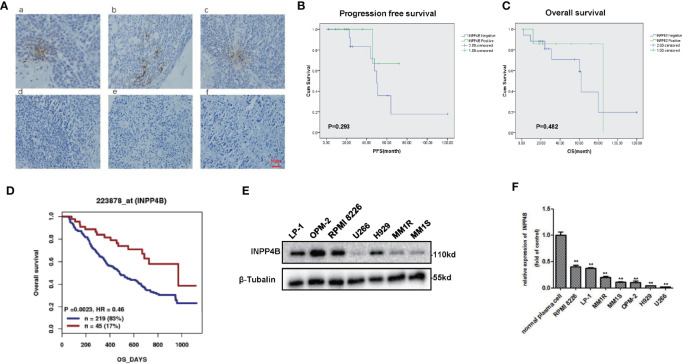
Expression of INPP4B in EMP samples and cell lines. **(A)** Immunohistochemical images of EMP tissue (focal positive: a, b, c; negative: d, e, f). Scar = 50 μm. **(B)** Patients stratified according to their INPP4B expression level (positive and negative) and correlation of INPP4B expression with progression-free survival (PFS). **(C)** Overall survival (OS) rate of patients with these extramedullary involvements. **(D)** Correlation of INPP4B expression and overall survival rate of MM patients in GSE7982 cohort. **(E)** INPP4B protein expression in various multiple myeloma cell lines. **(F)** INPP4B mRNA expression in various multiple myeloma cell lines. Data are plotted as mean ± SD from three independent experiments (**p < 0.01, vs. the control).

**Figure 3:**
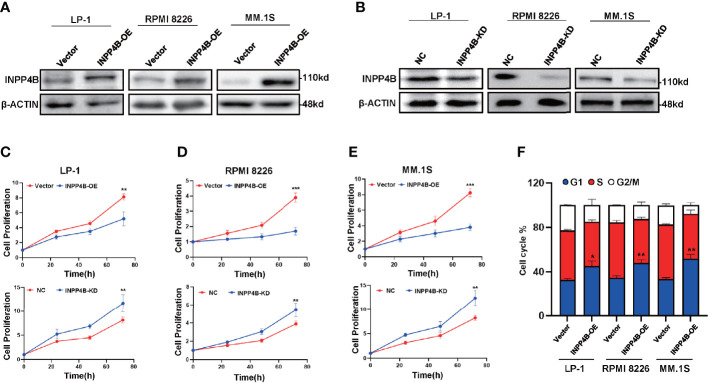
INPP4B overexpression inhibits the proliferation of MM cells *in vitro*. **(A)** Western blot shows the overexpression of INPP4B in LP-1, RPMI8226, and MM.1S cells infected with lentivirus carrying INPP4B. **(B)** Western blot assay reveals the efficiency of INPP4B knockdown by lentiviral-carrying infection of shRNA. The CCK8 assay to detect the effect of INPP4B knockdown (KD) and overexpression (OE) on cells proliferation in **(C)** LP-1, **(D)** RPMI8226, and **(E)** MM.1S cells. NC, non-target control. **(F)** Cell cycle assay shows that overexpression of INPP4B arrested MM cells at G0/G1 phase. Data were plotted as mean ± SD from three independent experiments (*p < 0.05, **p < 0.01, ***p < 0.001 vs. the control).

We further examined INPP4B levels in the extramedullary plasmacytoma (EMP) tissues and human MM cell lines. Immunohistochemistry (IHC) was used to assess the expression of INPP4B in tissues of EMP between January 2010 and December 2015. Seven extramedullary plasmacytoma patients expressed INPP4B reversible focal positive. Thirty-five extramedullary plasmacytoma patients expressed undetectable INPP4B (**Figure 2A**). The correlation between INPP4B expression and the clinical prognosis was analyzed, and we found that patients with INPP4B expression had a better prognosis than those without INPP4B expression, while there was no statistical difference on PFS (*p* = 0.293) and OS (*p* = 0.482) (**Figures 2B, C**). Notably, INPP4B expression predicted a positive association trend with patient’s overall survival in the cohort GSE9782 (**Figure 2D**). INPP4B protein expression in MM cell lines are shown in **Figure 2E**, respectively. Compared with normal human bone marrow plasma cells, the INPP4B mRNA expression level of seven MM cell lines was lower (**Figure 2F**). Taken together, these data suggest that decreased INPP4B may be correlated with MM malignancy. To further investigate how INPP4B influences MM malignancy, we successfully ectopically overexpressed INPP4B and silenced INPP4B in LP-1, RPMI 8226, and MM.1S cells, respectively (**Figures 3A, B**), after the optimized time point of overexpression and knockdown of INPP4B expression were confirmed (**Supplementary Figures S1A, B**). Notably, INPP4B overexpression significantly inhibited MM proliferation, while knockdown of INPP4B promoted proliferation of MM cells (**Figures 3C–E**). Moreover, we found that overexpression of INPP4B also arrested MM cells at G0/G1 phase (**Figure 3F**). These results indicate that INPP4B is a negative regulator of cell proliferation and cell cycle in MM cells.

**Figure 4:**
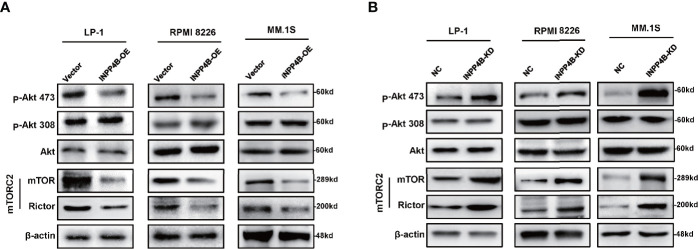
INPP4B inhibits MM cells growth *via* regulating the PI3K/Akt pathway. **(A)** Western blot analyzes the relative protein levels of Akt, p-Akt (thr308), p-Akt (ser473), rictor-mTOR complex (mTORC2) in LP-1, RPMI 8226, and MM.1S cells after lentiviral-carrying INPP4B overexpression for 48 h and **(B)** detections in LP-1, RPMI 8226, and MM.1S cells after lentiviral-carrying INPP4B knockdown for 48 h.

To further explore the potential mechanism of INPP4B inhibiting myeloma cells proliferation, we detected the effect of INPP4B on PI3K/Akt signaling pathways according to the previous reports (16). Since mTORC2 phosphorylates Akt at S473 (21), and RICTOR, a core component of mTORC2, acts as a key effector molecule of the PI3K-Akt pathway (22), we detected the key regulators, including Akt, p-Akt (thr308), p-Akt (ser473), and rictor-mTOR complex (mTORC2) in MM cells with INPP4B gain or loss of functions. We found that INPP4B overexpression decreased the phosphorylation of Akt at serine473 [p-Akt (ser473)], and INPP4B knockdown greatly increased p-Akt (ser473) in MM cell lines, under the condition that total protein levels of Akt showed no statistical difference. However, we did not find obvious changes in phosphorylation of Akt at threonine 308 (thr308). In addition, Western blot also showed that mTORC2 levels were remarkably suppressed accompanied by INPP4B overexpression, which is consistent with the previous report that mTORC2 normally enhances Akt signaling by phosphorylating its hydrophobic motif (Ser473) (23) (**Figures 4A, B**). These data suggest that INPP4B inhibits MM cells growth *via* regulating the PI3K/Akt/mTOR signaling pathway.

**Figure 5:**
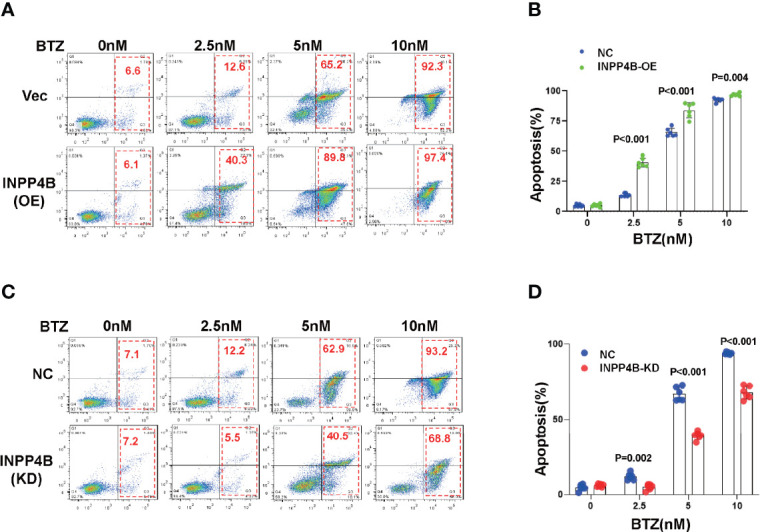
INPP4B promotes chemosensitivity to proteasome inhibitor in MM cells. **(A)** Flow cytometry assay showed the apoptosis of MM.1S cells infected with lentiviral-carrying INPP4B expressing vector and treated with different dosage of bortezomib (BTZ) for 48 h, and **(B)** showed the statistical analysis for three independent experiments. **(C)** Flow cytometry assay showed the apoptosis of MM.1S cells infected with lentiviral-carrying shRNA targeting INPP4B and treated with different dosage of bortezomib (BTZ) for 48 h, and **(B)** showed the statistical analysis for three independent experiments.

As low INPPB expression predicted poor outcomes in MM patients, we next evaluated whether manipulation of INPP4B expression could change sensitivity to chemotherapy reagent in the clinic, such as the first-line reagent widely applied in MM management, bortezomib. When the INPP4B expression was forcedly overexpressed in MM.1S cells, we found that MM cells became more sensitive to bortezomib at a dose-dependent manner, as evidenced by the elevated ratio of apoptotic cells (**Figure 5A**), and the differences were remarkable (**Figure 5B**). On the other hand, when HRP2 was knocked down in MM.1S cells, MM cells became more resistant to bortezomib treatment because the apoptotic ratio of MM cells was decreased significantly at a dose-dependent manner (**Figure 5C**), and the alterations were significant (**Figure 5D**). Collectively, these data strongly suggest that INPP4B is also a key regulator of sensitivity to chemo-sensitivity in MM cells.

## Discussion

In the present study, we elucidated the important role of INPP4B in inhibiting MM cell line proliferation and chemosensitivity *in vitro* and revealed that INPP4B was a low risk factor for MM patient in the clinic. Mechanistically, INPP4B exerted its role through inhibiting PI3K/Akt/mTORc2 signaling pathway. Our findings revealed a significant correlation between INPP4B expression and patient prognosis.

Previous findings have revealed dysregulation of PI3K/Akt signaling pathway in MM, and suppression of PI3K/Akt signaling could meliorate patient survival (11, 24). Inositol polyphosphate 4-phosphatase II encoded by the INPP4B genes are a kind of mammalian PI(3,4)P2 metabolizing 4-phosphatase enzymes. INPP4B converts phosphatidylinositol-3, 4-bisphosphate [PI(3,4)P2] to PI(3)P and has no effect on phosphatidylinositol-3.4.5-triphosphate [PI(3,4,5)P3]. PI(3,4)P2, like PI(3,4,5)P3, is necessary for the activation of Akt, enhancing tumor cell growth; therefore, INPP4B was hypothesized to be a tumor suppressor protein like PTEN, which dephosphorylates PI(3,4,5)P3(6). Thus, INPP4B is anticipated to act as a tumor suppressor by antagonizing PI3K/Akt signaling (25). Actually, the tumor-promoting features of INPP4B have also been found in several cancers, and it seems that INPP4B plays complex roles in the pathogenesis of different tumors (13, 26). PI3K/Akt signaling pathway plays a vital role in MM formation and growth, while the role of INPP4B in MM is not known. In this study, we found that overexpression of INPP4B in MM cell lines resulted in increased proliferation and decreased p-Akt mainly at Ser473 residues. Ser473 of Akt is a target site of mTORC2 (27), and our data also indicated the mTORc2 level was also suppressed by INPP4B overexpression, indicating that INPP4B could antagonize the hyperactivation of oncogenic PI3K/Akt/mTOR signaling. Thus, our study provides evidence for the correlation between decreased INPP4B expression and poor prognosis in the clinic.

Nevertheless, the current study has several limitations. First, MM patients have very complicated genomic and epigenetic backgrounds, for example, MM patients with t(4;14) translocations harboring high level of a histone methyltransferase, NSD2, which catalyzes histone dimethylation at lysine 36 (H3K63me2) (28), and these aberrations play a crucial role in MM roles in MM pathogenesis. We only proved that INPP4B inhibited the PI3K/Akt signaling pathway in LP-1, RPMI8226, and MM.1S, but these cells have different backgrounds; further work is needed to identify whether INPP4B influences the epigenetic modulation of MM. Second, the low expression of INPP4B in MM patients was not investigated. For example, whether the *INPP4B* promoter region is hyper-methylated has not been defined. Besides, whether the stability of INPP4B protein is regulated by ubiquitination or SUMOylated is not known. Finding key factors influencing INPP4B expression may shed light on developing new targeting strategies, since we do not have INPP4B inhibitors so far. Third, to make our conclusion more convincing, the animal study is needed to evaluate the role of INPP4B *in vivo*, especially whether PI3K inhibitors are more efficient in MM with low INPP4B expression. Finally, the correlation between INPP4B expression and prognosis of EMP was not significant, which may be due to limited cases.

In summary, results from the current study suggest that decreased INPP4B expression correlates with poor prognosis. We further demonstrate that INPP4B inhibits the proliferation and chemosensitivity of myeloma cells *via* disturbing PI3K/Akt/mTOR signaling and exerts a tumor suppressor effect. INPP4B may therefore have potential effect as a biomarker for disease progression. A number of PI3K inhibitors are now in clinical trials, and loss of INPP4B expression may provide a marker for selecting patients who will respond to these drugs.

## Data Availability Statement

The original contributions presented in the study are included in the article/Supplementary Material. Further inquiries can be directed to the corresponding authors at zhiqiangliu@tmu.edu.cn.

## Ethics Statement

This research project was approved by the Ethics Committee of Tianjin Cancer Institute and Hospital. Written consent was obtained from each patient. All procedures performed in studies involving human participants were in accordance with the ethical standards of the institutional and/or national research committee and with the 1964 Helsinki declaration and its later amendments or comparable ethical standards. The patients/participants provided their written informed consent to participate in this study.

## Author Contributions

YW and ZC contributed to writing the manuscript. LC, QL, YX, and JW to performed the experiments and statistical analyses. SG, JM, and SL provided the patient samples and clinical statistics. YW and ZL were in charge of the design of the experiments. All authors contributed to the article and approved the submitted version.

## Funding

This study was supported by the National Natural Science Foundation of China (No. 82000216).

## Conflict of Interest

The authors declare that the research was conducted in the absence of any commercial or financial relationships that could be construed as a potential conflict of interest.

## Publisher’s Note

All claims expressed in this article are solely those of the authors and do not necessarily represent those of their affiliated organizations, or those of the publisher, the editors and the reviewers. Any product that may be evaluated in this article, or claim that may be made by its manufacturer, is not guaranteed or endorsed by the publisher.
