# Supplementary material for: Inositol Polyphosphate 4-Phosphatase Type II Is a Tumor Suppressor in Multiple Myeloma
Source: Front Oncol. 2022 Jan 5;11:785297. doi: 10.3389/fonc.2021.785297 (PMC8767114; doi:10.3389/fonc.2021.785297)

**Supplementary Figure 1**. **Time-dependent effect of INPP4B overexpression and knockdown using lentivirus carrying infections in MM.1S cell.**

Western blot shows the **(A)**overexpression and **(B)** knockdown of INPP4B in MM.1S cells infected with lentivirus carrying INPP4B expressing vector or shRNA targeting INPP4B at 0hr, 24hr, 48hr and 72hr after infection.


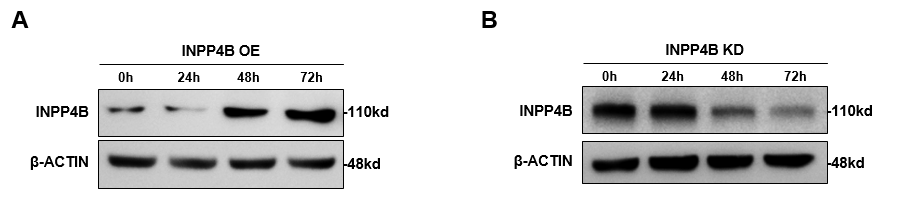

Supplement: Supplementary file 1 [file DataSheet_1.docx]
